# Predicting stroke in heart failure and reduced ejection fraction without atrial fibrillation^[Author-notes ehac487-FM2]^

**DOI:** 10.1093/eurheartj/ehac487

**Published:** 2022-08-26

**Authors:** Toru Kondo, Azmil H Abdul-Rahim, Atefeh Talebi, William T Abraham, Akshay S Desai, Kenneth Dickstein, Silvio E Inzucchi, Lars Køber, Mikhail N Kosiborod, Felipe A Martinez, Milton Packer, Mark Petrie, Piotr Ponikowski, Jean L Rouleau, Marc S Sabatine, Karl Swedberg, Michael R Zile, Scott D Solomon, Pardeep S Jhund, John J V McMurray

**Affiliations:** British Heart Foundation Cardiovascular Research Centre, University of Glasgow, 126 University Place, Glasgow G12 8TA, UK; Department of Cardiology, Nagoya University Graduate School of Medicine, Nagoya, Japan; Institute of Neuroscience and Psychology, College of Medical, Veterinary and Life Sciences, University of Glasgow, Glasgow, UK; British Heart Foundation Cardiovascular Research Centre, University of Glasgow, 126 University Place, Glasgow G12 8TA, UK; Division of Cardiovascular Medicine, The Ohio State University, OH, USA; Division of Cardiovascular Medicine, Brigham and Women’s Hospital, Boston, MA, USA; Department of Cardiology, Stavanger University Hospital, Stavanger, Norway; Section of Endocrinology, Yale University School of Medicine, New Haven, CT, USA; Department of Cardiology, Rigshospitalet Copenhagen University Hospital, Copenhagen, Denmark; Department of Cardiovascular Disease, Saint Luke's Mid America Heart Institute and University of Missouri-Kansas City, Kansas City, MO, USA; Universidad Nacional de Córdoba, International Society of Cardiovascular Pharmacotherapy, Córdoba, Argentina; Cardiovascular Science, Baylor Heart and Vascular Institute, Baylor University Medical Center, Dallas, TX, USA; British Heart Foundation Cardiovascular Research Centre, University of Glasgow, 126 University Place, Glasgow G12 8TA, UK; Department of Heart Disease, Wroclaw Medical University, Wroclaw, Poland; Department of Medicine, Montréal Heart Institute, Université de Montréal, Montréal, Quebec, Canada; TIMI Study Group, Division of Cardiovascular Medicine, Brigham and Women's Hospital, Boston, MA, USA; Department of Molecular and Clinical Medicine, University of Gothenburg, Gothenburg, Sweden; National Heart and Lung Institute, Imperial College London, London, UK; Department of Medicine, Medical University of South Carolina, Charleston, SC, USA; Division of Cardiovascular Medicine, Brigham and Women’s Hospital, Boston, MA, USA; British Heart Foundation Cardiovascular Research Centre, University of Glasgow, 126 University Place, Glasgow G12 8TA, UK; British Heart Foundation Cardiovascular Research Centre, University of Glasgow, 126 University Place, Glasgow G12 8TA, UK

**Keywords:** Heart failure, Stroke, Atrial fibrillation, Natriuretic peptides, Risk factors

## Abstract

**Aims:**

Patients with heart failure with reduced ejection fraction (HFrEF) are at significant risk of stroke. Anticoagulation reduces this risk in patients with and without atrial fibrillation (AF), but the risk-to-benefit balance in the latter group, overall, is not favourable. Identification of patients with HFrEF, without AF, at the highest risk of stroke may allow targeted and safer use of prophylactic anticoagulant therapy.

**Methods and results:**

In a pooled patient-level cohort of the PARADIGM-HF, ATMOSPHERE, and DAPA-HF trials, a previously derived simple risk model for stroke, consisting of three variables (history of prior stroke, insulin-treated diabetes, and plasma *N*-terminal pro-B-type natriuretic peptide level), was validated. Of the 20 159 patients included, 12 751 patients did not have AF at baseline. Among patients without AF, 346 (2.7%) experienced a stroke over a median follow up of 2.0 years (rate 11.7 per 1000 patient-years). The risk for stroke increased with increasing risk score: fourth quintile hazard ratio (HR) 2.35 [95% confidence interval (CI) 1.60–3.45]; fifth quintile HR 3.73 (95% CI 2.58–5.38), with the first quintile as reference. For patients in the top quintile, the rate of stroke was 21.2 per 1000 patient-years, similar to participants with AF not receiving anticoagulation (20.1 per 1000 patient-years). Model discrimination was good with a C-index of 0.84 (0.75–0.91).

**Conclusion:**

It is possible to identify a subset of HFrEF patients without AF with a stroke-risk equivalent to that of patients with AF who are not anticoagulated. In these patients, the risk-to-benefit balance might justify the use of prophylactic anticoagulation, but this hypothesis needs to be tested prospectively.

## Structured Graphical Abstract

**Structured Graphical Abstract:**
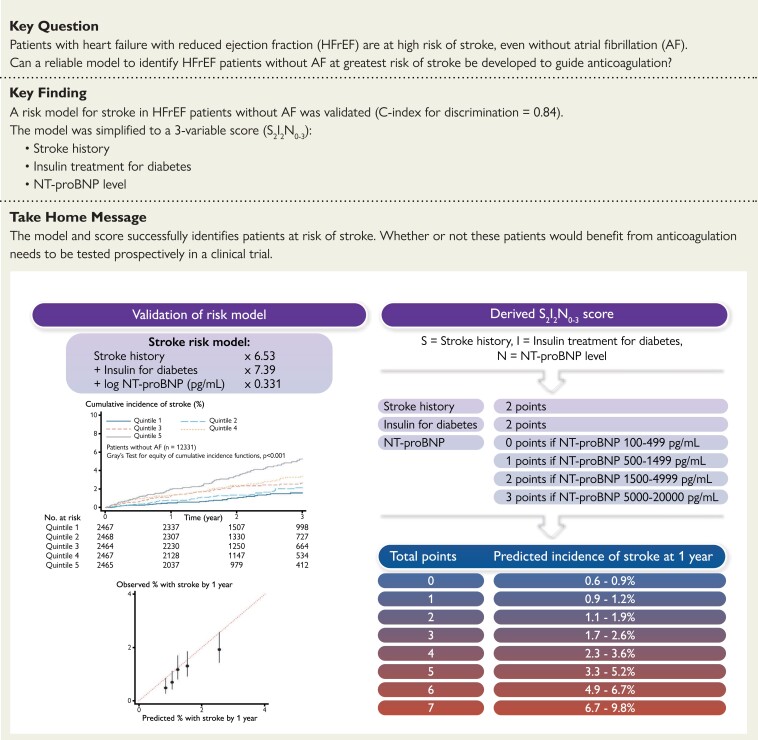
Validation of a risk score for stroke in heart failure with reduced ejection fraction. AF, atrial fibrillation; DM, diabetes mellitus; HFrEF, heart failure with reduced ejection fraction; NT-proBNP, *N*-terminal pro-B-type natriuretic peptide.

**See the editorial comment for this article ‘Stroke risk stratification in patients with heart failure and sinus rhythm’, by Dimitrios Sagris *et al*., https://doi.org/10.1093/eurheartj/ehac493.**

## Introduction

Stroke is a devastating complication of heart failure (HF) but is rarely considered when the clinical consequences of HF are described.^1^ If stroke is discussed in HF, it is usually in the context of associated atrial fibrillation (AF).^2–6^ However, stroke also occurs in HF patients without documented AF, although most older reports did not differentiate between individuals with and without AF. In a previous analysis of pooled data from large two trials, CORONA and GISSI-HF,^7,8^ we reported that the incidence of stroke in patients with HF and reduced ejection fraction (HFrEF) without AF was 11.1 per 1000 patient-years.^9^ However, CORONA and GISSI-HF were completed in 2007/2008 and may not have captured the most contemporary incidence of stroke in patients with HF.

More recently, the COMMANDER-HF trial showed that rivaroxaban reduced the incidence of stroke in patients with HFrEF in sinus rhythm.^10^ Unfortunately, because the overall incidence of stroke in the trial was relatively low, the absolute reduction in stroke was smaller than the absolute increase in major haemorrhage. However, it may be possible to identify a subgroup of patients at sufficiently high risk of stroke in which prophylactic anticoagulation has a favourable benefit-to-risk balance.

Previously, in a risk model developed using the CORONA and GISSI-HF data set, we found that patients in sinus rhythm in the upper tertile of risk had an incidence of stroke similar to that in patients with AF. This risk model for stroke consisted of just three variables: history of a previous stroke, insulin-treated diabetes, and plasma *N*-terminal pro-B-type natriuretic peptide (NT-proBNP) concentration.^9^ However, the model was not externally validated because no data set with NT-proBNP measurements was available at that time.

Therefore, we examined pooled patient-level data from three contemporary trials enrolling patients with HFrEF in whom NT-proBNP levels were measured at baseline: the Prospective comparison of angiotensin receptor neprilysin inhibitor with angiotensin-converting enzyme inhibitor to Determine Impact on Global Mortality and morbidity in Heart Failure trial (PARADIGM-HF, NCT01035255),^11^ the Aliskiren Trial to Minimize Outcomes in Patients with Heart Failure (ATMOSPHERE, NCT00853658),^12^ and the Dapagliflozin and Prevention of Adverse-outcomes in Heart Failure trial (DAPA-HF, NCT03036124).^13^ We aimed to evaluate the current incidence of stroke in patients with HFrEF without AF receiving modern pharmacological therapy and to validate our stroke prediction model in these contemporary HFrEF cohorts.

## Methods

### Study patients

The design and primary results of PARADIGM-HF, ATMOSPHERE, and DAPA-HF are published.^11–16^ Briefly, PARADIGM-HF included 8399 patients in New York Heart Association (NYHA) Functional Classes II–IV, previously treated with an angiotensin-converting enzyme inhibitor or angiotensin receptor blocker with a left-ventricular ejection fraction (LVEF) ≤40% (later changed to ≤35% by amendment), and a plasma NT-proBNP ≥600 pg/mL (or BNP ≥150 pg/mL) or NT-proBNP ≥400 pg/mL (or BNP ≥100 pg/mL) if hospitalized for HF within the last 12 months.^11,14^ Patients were also required to be treated with beta-blocker (if tolerated) and mineralocorticoid receptor antagonist therapy (if indicated). Patients entered a single-blind run-in period of 2 weeks treatment with enalapril 10 mg twice daily followed by a period of 2–4 weeks of treatment with sacubitril/valsartan 49/51 mg twice daily, increasing to 97/103 mg twice daily. Thereafter, patients were randomly assigned in a 1:1 ratio to double-blind treatment with either sacubitril/valsartan 97/103 mg twice daily or matching enalapril 10 mg twice daily. The primary outcome was the composite of cardiovascular death or HF hospitalization. The first patient entered the run-in period on 8 December 2009 and the median follow up was 27 months.

ATMOSPHERE included 7016 patients in NYHA Functional Classes II–IV with an LVEF ≤35%. Participants were also required to have an NT-proBNP concentration ≥600 pg/mL (or a BNP concentration ≥150 pg/mL), or an NT-proBNP concentration ≥400 pg/mL (or a BNP concentration ≥100 pg/mL) if they had been hospitalized for HF within the previous 12 months.^12,15^ Participants must have been receiving stable doses of an angiotensin-converting enzyme inhibitor (equivalent to at least 10 mg of enalapril daily) and of a beta-blocker at the time of enrolment. Patients entered a single-blind run-in phase involving 1–4 weeks of treatment with enalapril 5 mg twice daily, followed by 2–4 weeks of enalapril 10 mg twice daily. Patients then entered the second part of the run-in phase, during which they received aliskiren 150 mg once daily in addition to enalapril 5 or 10 mg twice daily. Thereafter, patients were assigned in a 1:1:1 ratio, to double-blind treatment with either the combination of enalapril 5 or 10 mg twice daily and aliskiren 150 mg once daily, aliskiren 150 mg once daily, or enalapril 5 or 10 mg twice daily. Two weeks after randomization, the dose of aliskiren was increased to 300 mg once daily in the combination-therapy group and the aliskiren group, with sham adjustment in the enalapril group. The primary outcome was a composite of death from cardiovascular causes or first hospitalization for HF. The first patient entered the run-in period on 13 March 2009 and the median follow up was 36.6 months.

DAPA-HF included 4744 patients in NYHA Functional Classes II–IV with an LVEF ≤40% who investigators considered optimally treated with pharmacological and device therapy for HF.^13,16^ Participants were also required to have an NT-proBNP concentration ≥600 pg/mL (≥400 pg/mL if hospitalized for HF within the previous 12 months). Patients with AF/atrial flutter were required to have an NT-proBNP level ≥900 pg/mL, irrespective of history of HF hospitalization. There was no run-in period and patients were randomly assigned in a 1:1 ratio to double-blind treatment with either dapagliflozin 10 mg once daily or a matching placebo. The primary outcome was the composite of cardiovascular death or a worsening HF event. The first patient was randomized on 15 February 2017 and the median follow up was 18.2 months.

Each trial was approved by local Ethics Committees and patients provided written informed consent.

### Incidence and cause of stroke

The occurrence and type of stroke were centrally adjudicated in each trial by a clinical events committee.^11–16^ Stroke events needed to meet the following criteria: (i) a rapid onset of a focal/global neurological deficit, (ii) duration of a focal/global neurological deficit ≥24 or <24 h, if the symptom improved due to pharmacologic or non-pharmacologic treatment or if available brain imaging clearly documented a new haemorrhage or infarct or if the neurological deficit resulted in death. Stroke events were adjudicated by two trained physician reviewers. If there was a major disagreement between the two reviewers, the endpoint committee met to discuss the case and made a final adjudication. We conducted sensitivity analyses including only strokes considered to have an ischaemic cause (including ischaemic stroke with haemorrhagic conversion); these analyses did not include strokes adjudicated as caused by a primary intracranial haemorrhage, or strokes which had an unknown cause or where neuroimaging was not performed.

### New-onset atrial fibrillation

The time to new onset of AF was a secondary outcome in PARADIGM-HF and ATMOSPHERE and a prespecified exploratory outcome in DAPA-HF. Information on the occurrence of AF was collected in each trial using a specific case report form.

### NT-proBNP evaluation

As described previously, NT-proBNP was measured using the same assay (Roche Elecsys) in all three trials using samples collected at baseline, before randomization, in a central laboratory.^17,18^

### Statistical methods

Patients with AF were defined as those with either AF confirmed on their baseline electrocardiogram or a prior history of AF (data regarding AF on electrocardiogram were missing in 133 cases in PARADIGM-HF, 5 cases in ATMOSPHERE, and 7 cases in DAPA-HF). The remaining patients were defined as those ‘without AF’. Descriptive statistics were used to describe the full cohort and to compare these two subgroups, using mean ± standard deviation, median [inter-quartile range (IQR)] for continuous variables, or number (percentage) for categorical variables. We also compared the baseline characteristics of patients who developed stroke during the trial and those without. All analyses were evaluated with complete-case analysis.

The incidence rate of stroke (per 1000 patient-years) was calculated during the trial follow-up period and compared among the aforementioned subgroups. Cumulative incidence functions (CIFs) of stroke occurrences were estimated accounting for competing risk of death.^19,20^ To satisfy the assumption of the independence of stroke events, the first event in a patient after randomization was assessed in the analysis.

We applied the previously published risk model for stroke derived from CORONA/GISSI-HF^9^ to patients with HFrEF without AF (derived from 206 strokes in 6054 patients) to the pooled data in patients without AF from the three contemporary HFrEF trials. The risk score was calculated by the equation: (history of previous stroke) × 6.53 + (insulin-treated diabetes) × 7.39 + [plasma NT-proBNP measurement at baseline (pmol/L) (in logarithmic transformation)] × 2.80. The coefficients from the previously published model were used to calculate an individual patient’s risk score for stroke. The unit of pg/mL of NT-proBNP was converted to pmol/L: 1 pg/mL = 0.1182 pmol/L. A total of 420 patients with a missing value on NT-proBNP were excluded from the model calculation. Cox proportional hazard model was performed to compute the hazard ratios (HRs) and 95% confidence interval (CI) of quintiles of risk score. Cumulative incidence function for stroke was estimated using competing risk technique^19,20^ according to quintiles of risk score.

To be consistent with our previous publication,^9^ we compared each model’s discrimination ability using estimates of the overall C-index for the risk model according to the method of Pencina and D’Agostino,^21^ as outlined by Liu *et al*.^22^ This method accounts for the survival times and censoring and is more appropriate for assessing the discriminatory performance of survival models than the previously used Harrell’s C-statistic.^23^ It may therefore give a different result to the Harrell’s C-statistic depending on the survival distribution. To allow comparison with the prior literature (published before the development and implementation of the overall C-index in statistical packages), we also present the traditional Harrell’s C-statistic.^24^ Model calibration and ability to separate populations of patients into risk groups were assessed by observing predicted vs. observed outcomes in quintiles.

We also conducted sensitivity analyses using ischaemic stroke as an outcome, with other stroke aetiologies censored (Sensitivity Analysis 1), and analyses excluding patients using an anticoagulant at baseline, with occurrence of AF and initiation of anticoagulant therapy as censoring events (Sensitivity Analysis 2).

Finally, based on the risk model, a simple score (S_2_I_2_N_0–3_) was created by assigning points to each component of the model. We assigned points based on NT-proBNP cutoffs that are easy to use in clinical practice, point totals that minimized the overlap in predicted incidence rates, and point totals with good discrimination and calibration. We reviewed a number of potential combinations of points and their respective incidence rates to derive the best score. All analyses were undertaken using SAS version 9.3 (SAS Institute, Inc., Cary, NC, USA), STATA version 17.0 (Stata Corp., College Station, TX, USA), and R version 4.1.2.

## Results

Of the 8399 patients in PARADIGM-HF, 3116 had either AF on their baseline electrocardiogram or a history of AF. The corresponding numbers were 2428 of the 7016 patients in ATMOSPHERE, and 1864 of the 4744 patients in DAPA-HF. This generated a total of 7408 patients with AF and 12 751 patients without AF in the pooled data set.

### Baseline characteristics

#### Patients with and without atrial fibrillation

The baseline demographics of patients with and without AF are shown in Supplementary material online, *Table S1*. Briefly, patients without AF were younger and had a lower LVEF and lower median plasma NT-proBNP level than patients with AF. There were also several differences in co-morbidity, notably in a history of myocardial infarction and hypertension with the former more common and the latter less frequent in patients without AF (compared with those with AF). The proportion of patients with diabetes was similar, but slightly more patients without AF were treated with insulin for their diabetes (compared with those with AF). There were also notable differences in medical therapy, particularly in the use of antiplatelet therapy (67.1% of patients without AF vs. 35.6% in those with AF) and anticoagulant treatment (12.5 vs. 69.8%).

#### Patients without atrial fibrillation, with and without stroke, during follow up

The baseline characteristics of patients without AF, according to whether or not they experienced a stroke after randomization are shown in *Table 1*. Patients without AF who experienced a stroke did not differ in age or from those who did not have a stroke and the proportion of men and women did not differ between those who did and did not develop stroke. Patients who experienced a stroke had higher blood pressure than those who did not. Patients developing stroke also had higher NT-proBNP levels compared with those who did not experience a stroke but had a similar LVEF. Patients experiencing a stroke were also more likely to have a history of prior stroke, hypertension, and diabetes (including insulin-treated diabetes) but kidney function did not differ between patients experiencing a stroke and those who did not. Patients who developed a stroke had a shorter duration of diagnosed HF than those who did not experience a stroke. Compared with this validation cohort, patients in the derivation cohort were older (69 years), had a similar prevalence of females, had a lower prevalence of hypertension (57%) but a similar prevalence of history of stroke and insulin-treated diabetes, and had a lower level of NT-proBNP (1023 pg/mL).^9^

**Table 1:** Baseline characteristics according to the occurrence of stroke during follow up in patients without atrial fibrillation

	All patients without AF (*n* = 12 751)	Patients without AF		*P*-Value
		No stroke (*n* = 12 405)	Stroke (*n* = 346)	
**Demographics, n (%)**				
Age, years	62.2 ± 11.7	62.2 ± 11.7	63.1 ± 11.5	0.15
≥65	5677 (44.5)	5529 (44.6)	148 (42.8)	0.51
≥75	1944 (15.2)	1884 (15.2)	60 (17.3)	0.27
**Race**				0.36
White	7475 (58.6)	7256 (58.5)	219 (63.3)	
Black	580 (4.5)	566 (4.6)	14 (4.0)	
Asian	3545 (27.8)	3459 (27.9)	86 (24.9)	
Other	1151 (9.0)	1124 (9.1)	27 (7.8)	
Female sex	2971 (23.3)	2889 (23.3)	82 (23.7)	0.86
**NYHA class**				0.49
I	298 (2.3)	288 (2.3)	10 (2.9)	
II	9016 (70.7)	8783 (70.8)	233 (67.3)	
III	3298 (25.9)	3198 (25.8)	100 (28.9)	
IV	132 (1.0)	129 (1.0)	3 (0.9)	
**Duration of heart failure**				0.011
≤1 year	4270 (33.5)	4176 (33.7)	94 (27.2)	
>1 year	8477 (66.5)	8225 (66.3)	252 (72.8)	
LVEF, %	28.9 ± 6.3	28.9 ± 6.3	28.8 ± 6.7	0.78
**Baseline vital signs**				
Body mass index, kg/m^2^	27.3 ± 5.5	27.3 ± 5.5	27.5 ± 5.1	0.44
Blood pressure, mmHg				
Systolic	122 ± 17	122 ± 17	125 ± 19	<0.001
Diastolic	74 ± 10	73 ± 10	75 ± 11	0.011
Pulse pressure	48 ± 13	48 ± 13	50 ± 15	0.024
Heart rate, b.p.m.	71 ± 11	71 ± 11	71 ± 12	0.98
**Laboratory measurements**				
Serum creatinine, µmol/L	95.6 ± 26.8	95.6 ± 26.8	96.5 ± 27.1	0.56
NT-proBNP, pg/mL, median (IQR)	1243 (704–2460)	1239 (700–2453)	1428 (821–2691)	0.011
**Medical history, n (%)**				
Coronary heart disease	7229 (56.7)	7021 (56.6)	208 (60.1)	0.19
Myocardial infarction	5911 (46.4)	5739 (46.3)	172 (49.7)	0.20
Angina pectoris	3149 (24.7)	3047 (24.6)	102 (29.5)	0.036
CABG or PCI	4521 (35.5)	4395 (35.4)	126 (36.4)	0.71
Hypertension	8182 (64.2)	7926 (63.9)	256 (74.0)	<0.001
Diabetes mellitus	4364 (34.2)	4230 (34.1)	134 (38.7)	0.073
Insulin-treated diabetes	1143 (9.0)	1102 (8.9)	41 (11.8)	0.057
Stroke	873 (6.8)	815 (6.6)	58 (16.8)	<0.001
Carotid artery disease^a^	523 (4.1)	504 (4.1)	19 (5.5)	0.19
Peripheral arterial disease^b^	737 (5.8)	721 (5.8)	16 (4.6)	0.35
Current smoker	1997 (15.7)	1940 (15.6)	57 (16.5)	0.67
**Treatments at randomization, n (%)**				
Diuretic	10 345 (81.1)	10 071 (81.2)	274 (79.2)	0.35
Digitalis	2675 (21.0)	2606 (21.0)	69 (19.9)	0.63
Beta-blocker	11 917 (93.5)	11 600 (93.5)	317 (91.6)	0.16
Mineralocorticoid receptor antagonist	6712 (52.6)	6550 (52.8)	162 (46.8)	0.028
Lipid lowering therapy	7760 (60.9)	7553 (60.9)	207 (59.8)	0.69
Antiplatelet therapy	8555 (67.1)	8313 (67.0)	242 (69.9)	0.25
Aspirin	7887 (61.9)	7659 (61.7)	228 (65.9)	0.12
ADP receptor inhibitor	2273 (17.8)	2221 (17.9)	52 (15.0)	0.17
Anticoagulant therapy	1598 (12.5)	1555 (12.5)	43 (12.4)	0.95
Any antithrombotic (antiplatelet or anticoagulant therapy)	9586 (75.2)	9315 (75.1)	271 (78.3)	0.17
Implantable cardioverter-defibrillator	2124 (16.7)	2072 (16.7)	52 (15.0)	0.41
Cardiac resynchronization therapy	743 (5.8)	720 (5.8)	23 (6.6)	0.51

Data are presented as mean ± standard deviation, median (IQR), or number (percentage). NYHA class was missing in 7 cases, duration of heart failure 4 cases, LVEF 1 case, body mass index 18 cases, blood pressure 1 case, heart rate 1 case, serum creatinine 104 cases, and *N*-terminal pro-B-type natriuretic peptide 420 cases.

ADP, adenosine diphosphate; AF, atrial fibrillation; CABG, coronary artery bypass graft; IQR, inter-quartile range; NT-proBNP, *N*-terminal pro-B-type natriuretic peptide; NYHA, New York Heart Association; LVEF, left-ventricular ejection fraction; PCI, percutaneous coronary intervention.

^a^ Carotid arterial disease is defined as the presence of carotid artery stenosis, previous history of carotid artery revascularization in the PARADIGM-HF and ATMOSPHERE, and the presence of carotid artery stenosis in the DAPA-HF.

b Peripheral arterial disease is defined as the presence of intermittent claudication, previous history of lower limb revascularization or lower limb stenosis documented by imaging in the PARADIGM-HF and ATMOSPHERE, and as the peripheral artery occlusive disease in the DAPA-HF.

Patients’ characteristics according to the occurrence of stroke for all patients and according to the use of anticoagulant therapy for patients with or without AF are shown in the Supplementary material online, *Tables S2 and S3*.

### Rates of stroke in PARADIGM-HF, ATMOSPHERE, and DAPA-HF

The median follow-up time in all patients in the pooled analysis was 2.0 (IQR: 1.4–3.1) years and 590 (2.9%) of the 20 159 patients experienced a stroke (12.7 per 1000 patient-years). In PARADIGM-HF, 219 patients experienced a stroke (11.9 per 1000 patient-years), in ATMOSPHERE, 283 patients (13.3 per 1000 patient-years), and in DAPA-HF, 88 patients (13.0 per 1000 patient-years).

#### Patients with atrial fibrillation

**Figure 1:**
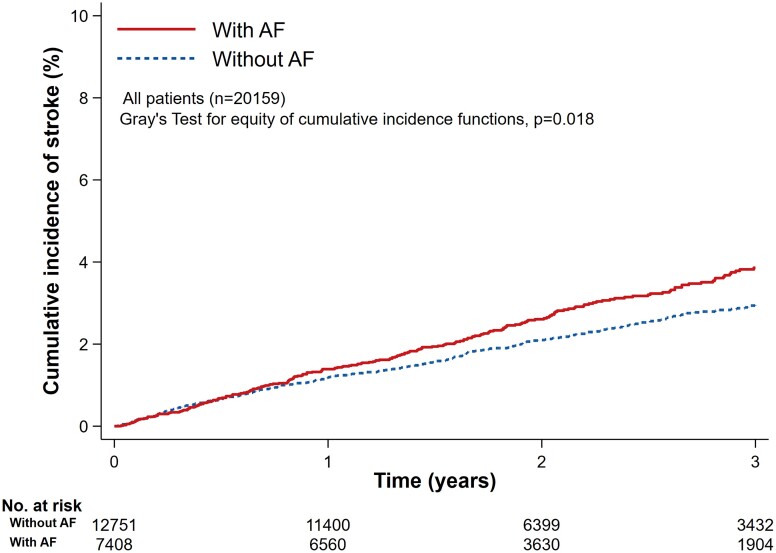
Cumulative incidence function plot for stroke by atrial fibrillation status at baseline (with death as a competing risk).

The median follow-up time in patients with AF was 2.0 (IQR: 1.4–3.1) years and 244 (3.3%) of these 7408 patients experienced a stroke (14.5 per 1000 patient-years). The 1-, 2-, and 3-year CIF rates of stroke were 1.4% (95% CI: 1.2–1.7), 2.6% (95% CI: 2.3–3.0), and 3.9% (95% CI: 3.5–4.4), respectively (*Figure 1*). The rate for stroke in patients treated with an anticoagulant was 12.1 per 1000 patient-years and in those not treated it was 20.1 per 1000 patient-years. In patients treated with an anticoagulant, the 1-, 2-, and 3-year CIF rates of stroke were 1.2% (95% CI: 0.9–1.5), 2.2% (95% CI: 1.9–2.7), and 3.2% (95% CI: 2.7–3.9), respectively (see Supplementary material online, *Figure S1*); the corresponding CIF rates for patients not on anticoagulant therapy were 1.9% (95% CI: 1.5–2.6), 3.4 (95% CI: 2.7–4.3), and 5.2 (95% CI: 4.2–6.4), respectively (see Supplementary material online, *Figure S1*).

#### Patients without atrial fibrillation

The median follow-up time in patients without AF was 2.0 (IQR: 1.4–3.1) years and 346 (2.7%) of these 12 751 patients experienced a stroke (11.7 per 1000 patient-years). The 1-, 2-, and 3-year CIF rates of stroke were 1.2% (95% CI: 1.0–1.4), 2.1% (95%CI: 1.9–2.4), and 3.0% (95%CI: 2.6–3.3), respectively (*Figure 1*).

Among the 11 153 patients not receiving an anticoagulant at baseline, 303 experienced a stroke (11.7 per 1000 patient-years). Among the 1598 patients receiving an anticoagulant at baseline, 43 experienced a stroke, giving a rate of 11.5 per 1000 patient-years.

Among patients on anticoagulant therapy, the 1-, 2-, and 3-year CIF rates of stroke were 1.2% (95% CI: 1.0–1.5), 2.1% (95% CI: 1.8–2.4), and 3.0% (95% CI: 2.6–3.4), respectively. The corresponding rates in those not on anticoagulant therapy were 1.3% (95% CI: 0.9–2.0), 2.2% (95% CI: 1.6–3.1), and 3.0% (95% CI: 2.1–4.1).

#### Incident atrial fibrillation and rate of stroke

Among patients without AF at baseline, new AF (i.e. incident AF) was detected in 528 patients (4.1%). Of the 346 patients without AF who experienced a stroke, 14 patients (4.0%) developed new AF before the occurrence of their stroke; the number of patients with a stroke without preceding AF was 332. Overall, 26 patients (7.5%) with incident stroke had new AF found before or after their stroke.

### Validation of the stroke prediction model

**Figure 2:**
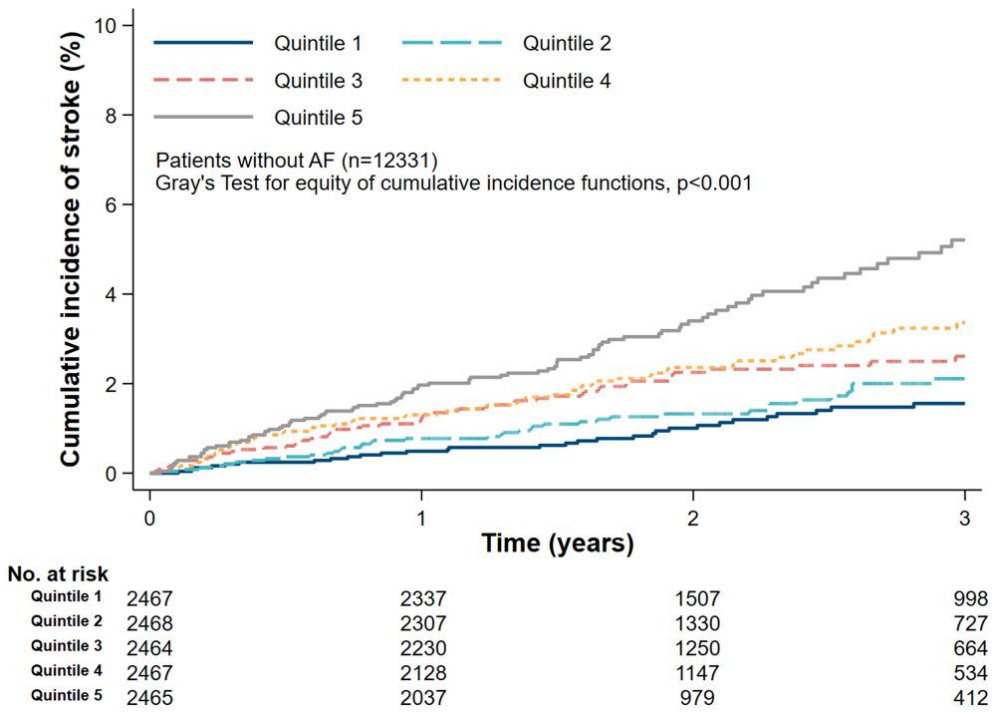
Cumulative incidence function plot for stroke by quintiles of the risk scores in patients without atrial fibrillation.

The distribution of the stroke-risk score is shown in Supplementary material online, *Figure S2*. *Figure 2* shows CIF plot for stroke with patients classified into five equally sized groups according to their risk score. The numbers of strokes in Quintiles 1, 2, 3, 4, and 5 were 41, 52, 64, 73, and 101, respectively. The 1-, 2- and 3-year CIF rates of stroke in the two higher risk quintiles were: Quintile 4, 1.3% (95% CI: 0.9–1.8), 2.4% (95% CI: 1.8–3.1), and 3.4% (95% CI: 2.6–4.4), respectively; and Quintile 5, 2.0% (95% CI: 1.5–2.6), 3.4% (95% CI: 2.7–4.3), and 5.2% (95% CI: 4.1–6.5), respectively. Patients in risk-Quintile 5 had an overall stroke rate of 21.2 per 1000 patient-years.

In Cox proportional hazard models, the risk of stroke increased as risk score increased (*Table 2*): Quintile 2, 1.48 (95% CI: 0.98–2.22); Quintile 3, 1.90 (95% CI: 1.28–2.81); Quintile 4, 2.35 (95% CI: 1.60–3.45); Quintile 5, 3.73 (95% CI: 2.58–5.38), with Quintile 1 as a reference.

**Table 2:** Validation of the stroke model in Cox proportional hazard model for patients without atrial fibrillation (*n* = 12 332)

	Number of stroke events (%)	Stroke rate (1000 patient-years)	Hazard ratio (95% CI)	*P*-value
Quintile 1	41 (1.7)	6.1	reference	
Quintile 2	52 (2.1)	8.7	1.48 (0.98–2.22)	0.063
Quintile 3	64 (2.6)	11.2	1.90 (1.28–2.81)	0.001
Quintile 4	73 (3.0)	13.7	2.35 (1.60–3.45)	<0.001
Quintile 5	101 (4.1)	21.2	3.73 (2.58–5.38)	<0.001

CI, confidence interval.

### Model calibration and discrimination

**Figure 3:**
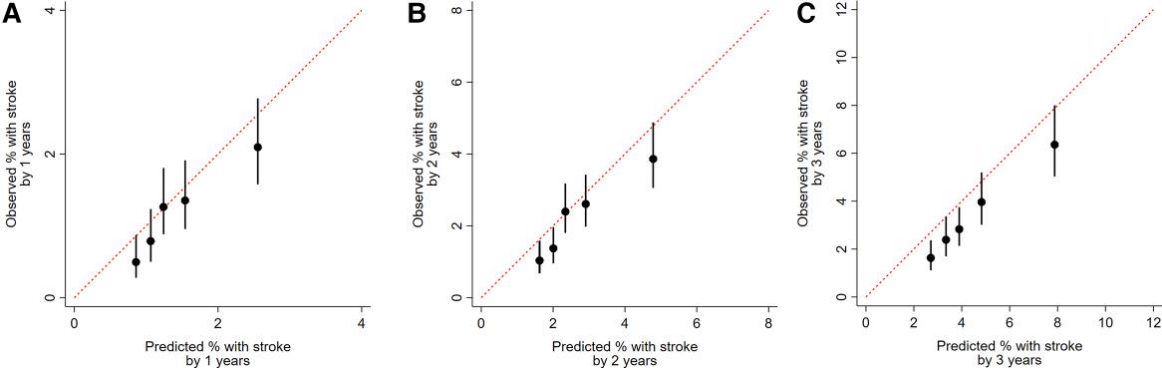
Comparison of observed and predicted strokes rates after 1–3 years for patients categorized by quintiles of risk score. Calibration plot for 1-year stroke event (*A*), 2-year stroke event (*B*), and 3-year stroke event (*C*). The circle indicates the observed rate and the vertical line indicates its range of 95% confidence interval. Each bar is ordered from left to right in descending order of quintiles of risk score. See the supplement for an explanation of how to calculate predicted strokes rates.

*Figure 3* shows calibration plots by comparing observed and predicted probabilities of a stroke at 1, 2, and 3 years, with the patients divided by quintiles. Model discrimination was good: the overall C-index was 0.84 (95% CI: 0.75–0.91).^21,22^ The overall C-index and Harrell’s C-statistics method are available in the online-only supplement (see Supplementary material online, *Table S4*).

### Sensitivity analysis

#### Sensitivity Analysis 1, using ischaemic stroke only as an outcome

Of the 346 strokes reported, 305 (88.2%) were considered to have an ischaemic cause (including ischaemic stroke with haemorrhagic conversion), 25 (7.2%) were due to a primary intracranial haemorrhage, and 16 (4.6%) had an unknown cause or did not have neuroimaging performed.

Consequently, the observed ischaemic stroke rates were a little lower than the model-based predicted stroke rates (for strokes of any cause), but model discrimination remained good with an overall C-index of 0.85 (95% CI: 0.75–0.92; see Supplementary material online, *Tables S5* and *S6* and *Figures S3* and *S4*).

#### Sensitivity Analysis 2, excluding patients receiving anticoagulant therapy at baseline with occurrence of atrial fibrillation, and initiation of anticoagulant therapy as censoring events

The observed stroke rates were marginally lower than the model-based predicted stroke rates (with only any stroke censored), and model discrimination remained good with an overall C-index of 0.84 (95% CI: 0.74–0.92; see Supplementary material online, *Tables S7* and *S8* and *Figures S5* and S6).

### The S_2_I_2_N_0–3_ score

The S_2_I_2_N_0–3_ score, created by assigning points to each component of the risk model, and the number of patients, observed and predicted incidence rate of stroke at 1 year, based on this score, are shown in *Table 3*. Patients with a maximum score of 7 points was expected to have about an 11 (7–16)-fold higher rate of stroke than individuals with a score of 0 points. The score discrimination was good with an overall C-index of 0.84 (95% CI: 0.76–0.92) (see Supplementary material online, *Table S9*).

**Table 3:** The S_2_I_2_N_0–3_ score

The S_2_I_2_N_0–3_ score is a simplified scoring method, created by assigning points to each component based on the risk model	
Stroke history	2 points
Insulin for DM	2 points
NT-proBNP	0 points if NT-proBNP 100–499 pg/mL
	1 points if NT-proBNP 500–1499 pg/mL
	2 points if NT-proBNP 1500–4999 pg/mL
	3 points if NT-proBNP 5000–20 000 pg/mL

Number of patients, observed and predicted incidence of stroke at 1 year according to the S_2_I_2_N_0–3_ score			
Total points	Number of patients	Observed stroke	Predicted incidence rate
0	1257 (10.4%)	0.47 per 100 patient-years	0.6–0.9%
1	4713 (38.9%)	0.91 per 100 patient-years	0.9–1.2%
2	3542 (29.2%)	1.25 per 100 patient-years	1.1–1.9%
3	1763 (14.5%)	1.83 per 100 patient-years	1.7–2.6%
4	597 (4.9%)	2.15 per 100 patient-years	2.3–3.6%
5	218 (1.8%)	3.12 per 100 patient-years	3.3–5.2%
6	30 (0.3%)	7.65 per 100 patient-years	4.9–6.7%
7	8 (0.1%)	–	6.7–9.8%

DM, diabetes mellitus; NT-proBNP, *N*-terminal pro-B-type natriuretic peptide.

## Discussion

Heart failure is thought to be an important cause of cardioembolic stroke, even in patients with sinus rhythm.^3–6^ Of the 55 incident ischaemic strokes in the aspirin group of the WARCEF trial, 22 (40%) were deemed to be definitely cardioembolic, 27 (49%) possibly cardioembolic, and 6 (11%) non-cardioembolic in origin.^25,26^ In the individual patient data set reported here, created from three recent clinical trials including 20 159 participants (and 590 strokes), we found that the incidence of stroke in patients without AF was 11.7 per 1000 patient-years. Although this was very similar to the rate previously reported in CORONA and GISSI-HF (11.1 per 1000 patient-years), it was lower than in the recent COMMANDER-HF trial which compared the effect of rivaroxaban with placebo on risk of atherothrombotic events, including stroke (stroke rate 16.2 per 1000 patient-years in the placebo group).^10^ However, patients enrolled in COMMANDER-HF were selected to be at high risk because of a recent episode of worsening HF and, as a result, 53.7% were in NYHA Class III/IV (compared with 26.9% in our data set). Following a protocol amendment, patients included in COMMANDER-HF were also required to have an NT-proBNP level ≥800 pg/mL and the median NT-proBNP at baseline was 2900 pg/mL (compared with 1243 pg/mL in our data set). As we have shown, both higher NYHA class and NT-proBNP levels are independent predictors of stroke.

However, in the present validation study, we confirmed that a simple model consisting of two clinical variables (history of stroke and diabetes treated with insulin) and NT-proBNP level successfully predicted the risk of stroke in HFrEF patients without AF (Structured *Graphical Abstract*). These variables likely reflect known risk factors for thrombus formation, including blood stasis, hypercoagulability, and endothelial damage, making the prognostic value of our model plausible, even in patients without AF.^3–6^ Our score allows stroke risk to be accurately and simply assessed in patients with HF without AF. It does not make sense to use the CHADS_2_ and CHA₂DS₂-VASc scores in this population, as each of these scores was developed in patients with AF.^27,28^ Moreover, CHADS_2_ and CHA₂DS₂-VASc include HF as a risk factor, and they were not designed to discriminate stroke risk in a population with HF without AF who have a substantial risk of stroke. CHADS_2_ and CHA₂DS₂-VASc were not derived and validated in HF patients without AF.

Using this risk model, we identified a fifth of patients with HFrEF and not in AF who had a similar incidence rate for stroke (21.2 per 1000 patient-years) as patients with AF not treated with an anticoagulant (20.1 per 1000 patient-years), and a higher incidence than patients with AF treated with an anticoagulant (12.1 per 1000 patient-years). In our prior analysis of the CORONA/GISSI-HF data set, patients in the highest risk category had an overall stroke rate of 22.9 per 1000 patient-years.^9^ The potential clinical importance of this finding is illustrated by the results of the WARCEF and COMMANDER-HF trials, both of which demonstrated that anticoagulant therapy reduced the risk of stroke in HFrEF patients in sinus rhythm.^10,26^ Specifically, in the most recent of these trials, COMMANDER-HF, the rate of stroke was reduced from 16.2 per 1000 patient-years in the placebo group to 10.8 per 1000 patient-years in the rivaroxaban group (HR 0.67, 95% CI 0.47–0.95).^29^ These trials support the hypothesis that stroke in such patients is often a result of cerebral embolization due to undiagnosed or new-onset AF, or embolization from the left ventricle or atrium in patients without AF.^30^ However, in both WARCEF and COMMANDER-HF, the reduction in stroke was less than the increase in the risk of major haemorrhage.^31^ Even with the non-vitamin K oral anticoagulant rivaroxaban (a direct Factor Xa inhibitor) used in COMMANDER-HF, there were 8 more patients per 1000 patient-years of treatment with major bleeding and 5 fewer patients with stroke per 1000 patient-years of treatment, compared with the placebo group. Therefore, a viable strategy to justify the use of anticoagulation in HFrEF patients in sinus rhythm to prevent stroke requires identification of patients at a higher absolute risk of stroke, reduction in risk of bleeding or both.^6^ The use of a risk score of the type described in the present article is one way in which anticoagulation could be targeted to patients at the highest risk of stroke, although the risk of haemorrhage needs to be taken into account at the same time. For example, if patients treated with an anticoagulant at baseline were excluded from those not in AF at highest risk (i.e. in the upper fifth of risk scores), the incidence of stroke was 21.0 per 1000 patient-years. Newer anticoagulants such as Factor XI inhibitors may carry a lower risk of haemorrhage.^32–37^

### Limitations

Our study had limitations as well as strengths. Our analysis was performed using data from three recent clinical trials. These trials had specific inclusion and exclusion criteria, and patients received excellent contemporary pharmacological treatment, better than described in many ‘real-world’ data sets. Hence, our findings may not be generalizable to all patients with HF, particularly patients with HF and preserved ejection fraction who were excluded from this analysis. In future studies, it would be interesting to validate our risk model in these patients and to compare it with other predictive models.^38,39^ Higher rates of stroke have been reported in some ‘real-world’ data sets, probably reflecting recent or current hospitalization and older age with accompanying predictive co-morbidities, including diabetes and history of stroke.^38,40–43^ We could not distinguish between Type 1 diabetes and Type 2 diabetes, so the specific risk of stroke in these two different conditions is unknown, although the large majority of HFrEF patients have Type 2 rather than Type 1 diabetes. While new-onset AF was a prespecified endpoint in each trial, systematic electrocardiographic monitoring was not undertaken, the incidence of AF reported was low, and short paroxysms of AF (or silent AF) may not have been detected, and the importance of these in relation to stroke occurrence cannot be estimated. However, a strategy of systematic electrocardiographic screening for AF would be labour intensive and expensive and probably not feasible in all patients with HFrEF. Moreover, such a strategy is potentially less efficacious than one using prophylactic anticoagulation in high-risk patients as stroke may occur at the time of or soon after the onset of AF and before anticoagulation can be employed.

## Conclusion

In conclusion, the present results validated our previously reported stroke-risk model and confirmed that it is possible to identify a subset of HFrEF patients without AF who have an incidence of stroke similar to that in patients with AF. This high-risk subset can be identified using two simple clinical variables and plasma NT-proBNP level. In these patients, the risk-to-benefit balance might justify the use of prophylactic anticoagulation to prevent stroke. This hypothesis needs to be tested in a prospective randomized controlled trial.

## Supplementary material

Supplementary material is available at *European Heart Journal* online.

## Supplementary Material

ehac487_Supplementary_Data

## Data Availability

Data from the DAPA-HF trial may be obtained following AstraZeneca’s data sharing policy described at https://astrazenecagrouptrials.pharmacm.com/ST/Submission/Disclosure. Data from the PARADIGM-HF and ATMOSPHERE trials may be obtained following Novartis’ data sharing policy described at www.clinicalstudydatarequest.com.
