# Supplementary material for: Predicting stroke in heart failure and reduced ejection fraction without atrial fibrillation
Source: Eur Heart J. 2022 Aug 26;43(42):4469–79. doi: 10.1093/eurheartj/ehac487 (PMC9637422; doi:10.1093/eurheartj/ehac487)
Supplement: ehac487_Supplementary_Data [file ehac487_supplementary_data.docx]

**SUPPLEMENTAL MATERIAL**

Online Supplement for manuscript entitled:

**Stroke in patients with heart failure and reduced ejection fraction without atrial fibrillation: validation of a risk model.**

**Authors:**

Toru Kondo MD, PhD; Azmil H Abdul-Rahim MSc, MD; Atefeh Talebi, PhD; William T Abraham, MD; Desai Akshay Suvas, M.D.,M.P.H; Kenneth Dickstein, MD; Silvio E Inzucchi, MD; Lars Køber, MD, DMSc; Mikhail N Kosiborod, MD; Felipe A Martinez, MD; Milton Packer, MD; Piotr Ponikowski, MD, PhD; Jean L Rouleau, MD; Marc S Sabatine, MD, MPH; Michael R Zile, MD; Scott D Solomon MD; Pardeep S Jhund MB ChB, MSc, PhD; John JV McMurray MD

**Supplementary Tables:** 1-9

**Supplementary Figures and Figure Legends:** 1-6

**Appendix:** Examples of calculation of risk score and 1 to 3-year incidence rate for stroke using the model, and S_2_I_2_N_0-3_ score

**SUPPLEMENTAL TABLES**

**Supplementary Table 1.** Baseline characteristics according to atrial fibrillation (AF) status at baseline.

|  | **All patients**  **(n=20159)** | **Without AF**  **(n=12751)** | **AF**  **(n=7408)** | **p-value** |
| --- | --- | --- | --- | --- |
| ***Demographics,* n (%)** |  |  |  |  |
| Age, year | 64.2±11.5 | 62.2±11.7 | 67.6±10.2 | <0.001 |
| ≥65 | 10414 (51.7) | 5677 (44.5) | 4737 (63.9) | <0.001 |
| ≥75 | 3921 (19.5) | 1944 (15.2) | 1977 (26.7) | <0.001 |
| Race |  |  |  | <0.001 |
| White | 13469 (66.8) | 7475 (58.6) | 5994 (80.9) |  |
| Black | 763 ( 3.8) | 580 ( 4.5) | 183 ( 2.5) |  |
| Asian | 4389 (21.8) | 3545 (27.8) | 844 (11.4) |  |
| Other | 1538 ( 7.6) | 1151 ( 9.0) | 387 ( 5.2) |  |
| Female sex | 4466 (22.2) | 2971 (23.3) | 1495 (20.2) | <0.001 |
| NYHA class |  | | | <0.001 |
| I | 390 ( 1.9) | 298 ( 2.3) | 92 ( 1.2) |  |
| II | 13558 (67.3) | 9016 (70.7) | 4542 (61.4) |  |
| III | 5957 (29.6) | 3298 (25.9) | 2659 (35.9) |  |
| IV | 240 ( 1.2) | 132 ( 1.0) | 108 ( 1.5) |  |
| Duration of heart failure, year |  |  |  | <0.001 |
| ≤ 1 year | 5982 (29.7) | 4270 (33.5) | 1712 (23.1) |  |
| > 1 year | 14173 (70.3) | 8477 (66.5) | 5696 (76.9) |  |
| LV Ejection Fraction, % | 29.5±6.3 | 28.9±6.3 | 30.4±6.1 | <0.001 |
| ***Baseline vital signs*** |  |  |  |  |
| Body mass index, kg/m^2^ | 27.9±5.6 | 27.3±5.5 | 28.9±5.5 | <0.001 |
| Blood pressure, mmHg |  |  |  |  |
| Systolic | 122±17 | 122±17 | 123±16 | <0.001 |
| Diastolic | 74±11 | 74±10 | 75±11 | <0.001 |
| Pulse pressure | 48±13 | 48±13 | 48±13 | 0.077 |
| Heart rate, beats/min | 72±12 | 71±11 | 74±13 | <0.001 |
| ***Laboratory measurements*** |  |  |  |  |
| Serum creatinine, µmol/l | 97.9±27.3 | 95.6±26.8 | 101.8±27.8 | <0.001 |
| NT-proBNP, pg/ml [(median (IQR)] | 1423 (792-2747) | 1243 (704-2460) | 1742 (1024-3103) | <0.001 |
| ***Medical history,* n (%)** |  |  |  |  |
| Coronary heart disease | 10991 (54.5) | 7229 (56.7) | 3762 (50.8) | <0.001 |
| Myocardial infarction | 8573 (42.5) | 5911 (46.4) | 2662 (35.9) | <0.001 |
| Angina pectoris | 5135 (25.5) | 3149 (24.7) | 1986 (26.8) | <0.001 |
| CABG or PCI | 6725 (33.4) | 4521 (35.5) | 2204 (29.8) | <0.001 |
| Hypertension | 13795 (68.4) | 8182 (64.2) | 5613 (75.8) | <0.001 |
| Diabetes mellitus | 6834 (33.9) | 4364 (34.2) | 2470 (33.3) | 0.20 |
| Insulin treated diabetes | 1737 ( 8.6) | 1143 ( 9.0) | 594 ( 8.0) | 0.021 |
| Stroke | 1683 ( 8.3) | 873 ( 6.8) | 810 (10.9) | <0.001 |
| Carotid artery disease * | 816 ( 4.0) | 523 ( 4.1) | 293 ( 4.0) | 0.61 |
| Peripheral arterial disease † | 1176 ( 5.8) | 737 ( 5.8) | 439 ( 5.9) | 0.67 |
| Current smoker | 2802 (13.9) | 1997 (15.7) | 805 (10.9) | <0.001 |
| ***Treatments at randomization,* n (%)** |  | | |  |
| Diuretic | 16769 (83.2) | 10345 (81.1) | 6424 (86.7) | <0.001 |
| Digitalis | 5668 (28.1) | 2675 (21.0) | 2993 (40.4) | <0.001 |
| Beta-blocker | 18801 (93.3) | 11917 (93.5) | 6884 (92.9) | 0.15 |
| Mineralocorticoid antagonist | 10643 (52.8) | 6712 (52.6) | 3931 (53.1) | 0.56 |
| Lipid lowering therapy | 11689 (58.0) | 7760 (60.9) | 3929 (53.0) | <0.001 |
| Antiplatelet therapy | 11195 (55.5) | 8555 (67.1) | 2640 (35.6) | <0.001 |
| Aspirin | 10,216 (50.7) | 7,887 (61.9) | 2,329 (31.4) | <0.001 |
| ADP receptor inhibitor | 2,862 (14.2) | 2,273 (17.8) | 589 ( 8.0) | <0.001 |
| Anticoagulant therapy | 6772 (33.6) | 1598 (12.5) | 5174 (69.8) | <0.001 |
| Any antithrombotic (antiplatelet or anti-coagulant therapy) | 16294 (80.8) | 9586 (75.2) | 6708 (90.6) | <0.001 |
| Implantable cardioverter-defibrillator | 3533 (17.5) | 2124 (16.7) | 1409 (19.0) | <0.001 |
| Cardiac resynchronization therapy | 1321 ( 6.6) | 743 ( 5.8) | 578 ( 7.8) | <0.001 |

Data are presented as mean±standard deviation, median (IQR), or number (percentage).

ADP indicates adenosine diphosphate; AF, atrial fibrillation; CABG, coronary artery bypass graft; IQR, inter-quartile range; NT-proBNP, N-terminal pro-B-type natriuretic peptide; NYHA, New York Heart Association; LV, left ventricular: PCI, percutaneous coronary intervention.

NYHA class was missing in 14 cases, duration of heart failure 4 cases, LV ejection fraction 1 case, body mass index 31 cases, blood pressure 2 case, heart rate 2 case, serum creatinine 179 cases, and N-terminal pro-B-type natriuretic peptide 680 cases.

* Carotid arterial disease is defined as the presence of carotid artery stenosis, previous history of carotid artery revascularization in the PARADIGM-HF and ATMOSPHERE, and the presence of carotid artery stenosis in the DAPA-HF.

† Peripheral arterial disease is defined as the presence of intermittent claudication symptom or previous history of lower limb revascularization or lower limb stenosis documented by imaging in the PARADIGM-HF and ATMOSPHERE, and as the peripheral artery occlusive disease in the DAPA-HF.

**Supplementary Table 2.** Baseline characteristics according to the occurrence of stroke

|  | **All patients**  **(n=20159)** | **No stroke**  **(n=19569)** | **Stroke**  **(n=590)** | **p-value** |
| --- | --- | --- | --- | --- |
| ***Demographics,* n (%)** |  |  |  |  |
| Age, year | 64.2±11.5 | 64.2±11.5 | 65.3±11.0 | 0.021 |
| ≥65 | 10414 (51.7) | 10105 (51.6) | 309 (52.4) | 0.72 |
| ≥75 | 3921 (19.5) | 3788 (19.4) | 133 (22.5) | 0.054 |
| Race |  |  |  | 0.49 |
| White | 13469 (66.8) | 13058 (66.7) | 411 (69.7) |  |
| Black | 763 ( 3.8) | 741 ( 3.8) | 22 ( 3.7) |  |
| Asian | 4389 (21.8) | 4271 (21.8) | 118 (20.0) |  |
| Other | 1538 ( 7.6) | 1499 ( 7.7) | 39 ( 6.6) |  |
| Female sex | 4466 (22.2) | 4310 (22.0) | 156 (26.4) | 0.011 |
| NYHA class |  | | | 0.003 |
| I | 390 ( 1.9) | 378 ( 1.9) | 12 ( 2.0) |  |
| II | 13558 (67.3) | 13195 (67.5) | 363 (61.5) |  |
| III | 5957 (29.6) | 5745 (29.4) | 212 (35.9) |  |
| IV | 240 ( 1.2) | 237 ( 1.2) | 3 ( 0.5) |  |
| Duration of heart failure, year |  |  |  | 0.013 |
| ≤ 1 year | 5982 (29.7) | 5834 (29.8) | 148 (25.1) |  |
| > 1 year | 14173 (70.3) | 13731 (70.2) | 442 (74.9) |  |
| LV Ejection Fraction, % | 29.5±6.3 | 29.5±6.3 | 29.7±6.3 | 0.48 |
| Diagnosis of AF | 7408 (36.7) | 7164 (36.6) | 244 (41.4) | 0.018 |
| History of AF | 7299 (36.2) | 7059 (36.1) | 240 (40.7) | 0.022 |
| Baseline ECG of AF | 4888 (24.4) | 4716 (24.3) | 172 (29.4) | 0.004 |
| ***Baseline vital signs*** |  |  |  |  |
| Body mass index, kg/m^2^ | 27.9±5.6 | 27.9±5.6 | 27.9±5.2 | 0.99 |
| Blood pressure, mmHg |  |  |  |  |
| Systolic | 122±17 | 122±17 | 125±18 | <0.001 |
| Diastolic | 74±11 | 74±11 | 76±11 | <0.001 |
| Pulse pressure | 48±13 | 48±13 | 49±14 | 0.038 |
| Heart rate, beats/min | 72±12 | 72±12 | 73±13 | 0.035 |
| ***Laboratory measurements*** |  |  |  |  |
| Serum creatinine, µmol/l | 97.9±27.3 | 97.9±27.3 | 98.1±26.5 | 0.83 |
| NT-proBNP, pg/ml [(median (IQR)] | 1423 (792-2747) | 1416 (787-2736) | 1638 (947-2943) | <0.001 |
| ***Medical history,* n (%)** |  |  |  |  |
| Coronary heart disease | 10991 (54.5) | 10656 (54.5) | 335 (56.8) | 0.26 |
| Myocardial infarction | 8573 (42.5) | 8313 (42.5) | 260 (44.1) | 0.44 |
| Angina pectoris | 5135 (25.5) | 4956 (25.3) | 179 (30.3) | 0.006 |
| CABG or PCI | 6725 (33.4) | 6536 (33.4) | 189 (32.0) | 0.49 |
| Hypertension | 13795 (68.4) | 13338 (68.2) | 457 (77.5) | <0.001 |
| Diabetes mellitus | 6834 (33.9) | 6635 (33.9) | 199 (33.7) | 0.93 |
| Insulin treated diabetes | 1737 ( 8.6) | 1677 ( 8.6) | 60 (10.2) | 0.17 |
| Stroke | 1683 ( 8.3) | 1584 ( 8.1) | 99 (16.8) | <0.001 |
| Carotid artery disease * | 816 ( 4.0) | 793 ( 4.1) | 23 ( 3.9) | 0.85 |
| Peripheral arterial disease † | 1176 ( 5.8) | 1140 ( 5.8) | 36 ( 6.1) | 0.78 |
| Current smoker | 2802 (13.9) | 2723 (13.9) | 79 (13.4) | 0.72 |
| ***Treatments at randomization,* n (%)** |  | | |  |
| Diuretic | 16769 (83.2) | 16287 (83.2) | 482 (81.7) | 0.33 |
| Digitalis | 5668 (28.1) | 5499 (28.1) | 169 (28.6) | 0.77 |
| Beta-blocker | 18801 (93.3) | 18259 (93.3) | 542 (91.9) | 0.17 |
| Mineralocorticoid antagonist | 10643 (52.8) | 10370 (53.0) | 273 (46.3) | 0.001 |
| Lipid lowering therapy | 11689 (58.0) | 11359 (58.0) | 330 (55.9) | 0.31 |
| Antiplatelet therapy | 11195 (55.5) | 10841 (55.4) | 354 (60.0) | 0.027 |
| Aspirin | 10,216 (50.7) | 9,886 (50.5) | 330 (55.9) | 0.010 |
| ADP receptor inhibitor | 2,862 (14.2) | 2,786 (14.2) | 76 (12.9) | 0.35 |
| Anticoagulant therapy | 6772 (33.6) | 6589 (33.7) | 183 (31.0) | 0.18 |
| Any antithrombotic (antiplatelet or anti-coagulant therapy) | 16294 (80.8) | 15803 (80.8) | 491 (83.2) | 0.13 |
| Implantable cardioverter-defibrillator | 3533 (17.5) | 3454 (17.7) | 79 (13.4) | 0.007 |
| Cardiac resynchronization therapy | 1321 ( 6.6) | 1285 ( 6.6) | 36 ( 6.1) | 0.65 |

Data are presented as mean±standard deviation, median (IQR), or number (percentage).

ADP indicates adenosine diphosphate; AF, atrial fibrillation; CABG, coronary artery bypass graft; IQR, inter-quartile range; NT-proBNP, N-terminal pro-B-type natriuretic peptide; NYHA, New York Heart Association; LV, left ventricular: PCI, percutaneous coronary intervention.

NYHA class was missing in 14 cases, duration of heart failure 4 cases, LV ejection fraction 1 case, body mass index 31 cases, blood pressure 2 case, heart rate 2 case, serum creatinine 179 cases, and N-terminal pro-B-type natriuretic peptide 680 cases.

* Carotid arterial disease is defined as the presence of carotid artery stenosis, previous history of carotid artery revascularization in the PARADIGM-HF and ATMOSPHERE, and the presence of carotid artery stenosis in the DAPA-HF.

† Peripheral arterial disease is defined as the presence of intermittent claudication symptom or previous history of lower limb revascularization or lower limb stenosis documented by imaging in the PARADIGM-HF and ATMOSPHERE, and as the peripheral artery occlusive disease in the DAPA-HF.

**Supplementary Table 3.** Baseline characteristics according to the use of anticoagulant

|  | **Patients without AF** | | |  | **Patients with AF** | |  |
| --- | --- | --- | --- | --- | --- | --- | --- |
|  | **Without anticoagulant**  **(n=11153)** | **With anticoagulant**  **(n=1598)** | |  | **Without anticoagulant**  **(n=2234)** | **With anticoagulant**  **(n=5174)** |  |
| ***Demographics,* n (%)** |  |  | |  |  |  |  |
| Age, year | 62.3±11.8 | 61.9±11.3 | | 0.29 | 67.3±10.7 | 67.7±9.9 | 0.13 |
| ≥65 | 5,005 (44.9) | 672 (42.1) | | 0.034 | 1,387 (62.1) | 3,350 (64.7) | 0.029 |
| ≥75 | 1,725 (15.5) | 219 (13.7) | | 0.067 | 618 (27.7) | 1,359 (26.3) | 0.21 |
| Race |  |  | | <0.001 |  |  | <0.001 |
| White | 6,335 (56.8) | 1,140 (71.3) | |  | 1,597 (71.5) | 4,397 (85.0) |  |
| Black | 514 ( 4.6) | 66 ( 4.1) | |  | 71 ( 3.2) | 112 ( 2.2) |  |
| Asian | 3,277 (29.4) | 268 (16.8) | |  | 400 (17.9) | 444 ( 8.6) |  |
| Other | 1,027 ( 9.2) | 124 ( 7.8) | |  | 166 ( 7.4) | 221 ( 4.3) |  |
| Female sex | 2,653 (23.8) | 318 (19.9) | | <0.001 | 524 (23.5) | 971 (18.8) | <0.001 |
| NYHA class |  | | 0.005 | |  | | <0.001 |
| I | 267 ( 2.4) | 31 ( 1.9) | |  | 42 ( 1.9) | 50 ( 1.0) |  |
| II | 7,829 (70.2) | 1,187 (74.3) | |  | 1,248 (56.0) | 3,294 (63.7) |  |
| III | 2,929 (26.3) | 369 (23.1) | |  | 878 (39.4) | 1,781 (34.4) |  |
| IV | 122 ( 1.1) | 10 ( 0.6) | |  | 62 ( 2.8) | 46 ( 0.9) |  |
| Duration of heart failure, year |  |  | | <0.001 |  |  | 0.043 |
| ≤ 1 year | 3,890 (34.9) | 380 (23.8) | |  | 550 (24.6) | 1,162 (22.5) |  |
| > 1 year | 7,259 (65.1) | 1,218 (76.2) | |  | 1,684 (75.4) | 4,012 (77.5) |  |
| LV Ejection Fraction, % | 29.1±6.2 | 27.6±6.9 | | <0.001 | 30.5±5.9 | 30.4±6.2 | 0.74 |
| ***Baseline vital signs*** |  |  | |  |  |  |  |
| Body mass index, kg/m^2^ | 27.3±5.5 | 27.7±5.4 | | 0.003 | 28.0±5.3 | 29.2±5.6 | <0.001 |
| Blood pressure, mmHg | 122±17 | 118±15 | | <0.001 |  |  |  |
| Systolic | 74±10 | 72±10 | | <0.001 | 123±17 | 123±16 | 0.085 |
| Diastolic | 49±13 | 46±12 | | <0.001 | 75±11 | 75±11 | 0.32 |
| Pulse pressure | 71±11 | 70±11 | | <0.001 | 49±13 | 48±13 | 0.002 |
| Heart rate, beats/min |  |  | |  | 73±13 | 74±14 | 0.30 |
| ***Laboratory measurements*** |  |  | |  |  |  |  |
| Serum creatinine, µmol/l | 95.1±26.8 | 99.2±26.6 | | <0.001 | 99.5±28.4 | 102.8±27.4 | <0.001 |
| NT-proBNP, pg/ml [(median (IQR)] | 1244 (703-2452) | 1238 (722-2513) | | 0.56 | 1699 (938-3167) | 1756 (1061-3077) | 0.056 |
| ***Medical history,* n (%)** |  |  | |  |  |  |  |
| Coronary heart disease | 6,281 (56.3) | 948 (59.3) | | 0.023 | 1,251 (56.0) | 2,511 (48.5) | <0.001 |
| Myocardial infarction | 5,112 (45.8) | 799 (50.0) | | 0.002 | 901 (40.3) | 1,761 (34.0) | <0.001 |
| Angina pectoris | 2,766 (24.8) | 383 (24.0) | | 0.47 | 760 (34.0) | 1,226 (23.7) | <0.001 |
| CABG or PCI | 3,885 (34.8) | 636 (39.8) | | <0.001 | 651 (29.1) | 1,553 (30.0) | 0.45 |
| Hypertension | 7,262 (65.1) | 920 (57.6) | | <0.001 | 1,699 (76.1) | 3,914 (75.6) | 0.71 |
| Diabetes mellitus | 3,880 (34.8) | 484 (30.3) | | <0.001 | 661 (29.6) | 1,809 (35.0) | <0.001 |
| Insulin treated diabetes | 1,005 ( 9.0) | 138 ( 8.6) | | 0.62 | 146 ( 6.5) | 448 ( 8.7) | 0.002 |
| Stroke | 670 ( 6.0) | 203 (12.7) | | <0.001 | 198 ( 8.9) | 612 (11.8) | <0.001 |
| Carotid artery disease * | 448 ( 4.0) | 75 ( 4.7) | | 0.20 | 104 ( 4.7) | 189 ( 3.7) | 0.042 |
| Peripheral arterial disease † | 619 ( 5.6) | 118 ( 7.4) | | 0.003 | 124 ( 5.6) | 315 ( 6.1) | 0.37 |
| Current smoker | 1,752 (15.7) | 245 (15.3) | | 0.70 | 278 (12.4) | 527 (10.2) | 0.004 |
| ***Treatments at randomization,* n (%)** |  | |  | |  | |  |
| Diuretic | 9,000 (80.7) | 1,345 (84.2) | | <0.001 | 1,803 (80.7) | 4,621 (89.3) | <0.001 |
| Digitalis | 2,324 (20.8) | 351 (22.0) | | 0.30 | 828 (37.1) | 2,165 (41.8) | <0.001 |
| Beta-blocker | 10,391 (93.2) | 1,526 (95.5) | | <0.001 | 2,032 (91.0) | 4,852 (93.8) | <0.001 |
| Mineralocorticoid antagonist | 5,770 (51.7) | 942 (58.9) | | <0.001 | 1,147 (51.3) | 2,784 (53.8) | 0.051 |
| Lipid lowering therapy | 6,752 (60.5) | 1,008 (63.1) | | 0.052 | 1,067 (47.8) | 2,862 (55.3) | <0.001 |
| Antiplatelet therapy | 7,988 (71.6) | 567 (35.5) | | <0.001 | 1,534 (68.7) | 1,106 (21.4) | <0.001 |
| Aspirin | 7,393 (66.3) | 494 (30.9) | | <0.001 | 1,384 (62.0) | 945 (18.3) | <0.001 |
| ADP receptor inhibitor | 2,130 (19.1) | 143 ( 8.9) | | <0.001 | 354 (15.8) | 235 ( 4.5) | <0.001 |
| Implantable cardioverter-defibrillator | 1,612 (14.5) | 512 (32.0) | | <0.001 | 293 (13.1) | 1,116 (21.6) | <0.001 |
| Cardiac resynchronization therapy | 550 ( 4.9) | 193 (12.1) | | <0.001 | 129 ( 5.8) | 449 ( 8.7) | <0.001 |

Data are presented as mean±standard deviation, median (IQR), or number (percentage).

ADP indicates adenosine diphosphate; AF, atrial fibrillation; CABG, coronary artery bypass graft; IQR, inter-quartile range; NT-proBNP, N-terminal pro-B-type natriuretic peptide; NYHA, New York Heart Association; LV, left ventricular: PCI, percutaneous coronary intervention.

NYHA class was missing in 14 cases, duration of heart failure 4 cases, LV ejection fraction 1 case, body mass index 31 cases, blood pressure 2 case, heart rate 2 case, serum creatinine 179 cases, and N-terminal pro-B-type natriuretic peptide 680 cases.

* Carotid arterial disease is defined as the presence of carotid artery stenosis, previous history of carotid artery revascularization in the PARADIGM-HF and ATMOSPHERE, and the presence of carotid artery stenosis in the DAPA-HF.

† Peripheral arterial disease is defined as the presence of intermittent claudication symptom or previous history of lower limb revascularization or lower limb stenosis documented by imaging in the PARADIGM-HF and ATMOSPHERE, and as the peripheral artery occlusive disease in the DAPA-HF.

**Supplementary Table 4.** Comparison of the model’s discrimination using the overall C-index and the traditional Harrell’s C-statistic.

|  | **Overall C-index*** (95%CI) | **Harrell’s C-statistic^†^** (95%CI) |
| --- | --- | --- |
| Stroke model | 0.84 (0.75-0.91) | 0.63 (0.60-0.66) |

* Overall C-index is calculated according to Pencina *et al* method ^21^, as outlined by Liu *et al* ^22^.

^†^ Harrell’s C-statistic is calculated using Harrell *et al* method^18^.

**Supplementary Table 5. *Sensitivity analysis 1***- Validation of stroke model in Cox proportional hazard model for patients without AF for outcome of ischaemic stroke (n=12332).

|  | **Number of stroke events (%)** | **Stroke rate (1000 patient-years)** | **Hazard ratio (95% CI)** | **p-value** |
| --- | --- | --- | --- | --- |
| Quintile 1 | 39 (1.6) | 5.8 | reference |  |
| Quintile 2 | 46 (1.9) | 7.7 | 1.36 (0.89-2.09) | 0.16 |
| Quintile 3 | 53 (2.2) | 9.2 | 1.64 (1.08-2.48) | 0.020 |
| Quintile 4 | 64 (2.6) | 12.0 | 2.14 (1.44-3.20) | <0.001 |
| Quintile 5 | 88 (3.6) | 18.5 | 3.35 (2.29-4.91) | <0.001 |

CI: confidence interval.

**Supplementary Table 6. *Sensitivity analysis 1***- Comparison of the model’s discrimination using the overall C-index and the traditional Harrell’s C-statistic for ischaemic stroke.

|  | **Overall C-index*** (95%CI) | **Harrell’s C-statistic^†^** (95%CI) |
| --- | --- | --- |
| Stroke model | 0.85 (0.75-0.92) | 0.63 (0.59-0.66) |

* Overall C-index is calculated according to Pencina *et al* method ^21^, as outlined by Liu *et al* ^22^.

^†^ Harrell’s C-statistic is calculated using Harrell *et al* method^11^.

**Supplementary Table 7. *Sensitivity analysis 2***- Validation of stroke model in Cox proportional hazard model for patients without AF and not receiving anticoagulant therapy at baseline, with occurrence of AF and initiation of anticoagulant therapy as censoring events (n=10781).

|  | **Number of stroke events (%)** | **Stroke rate (1000 patient-years)** | **Hazard ratio (95% CI)** | **p-value** |
| --- | --- | --- | --- | --- |
| Quintile 1 | 33 (1.5) | 6.0 | reference |  |
| Quintile 2 | 39 (1.8) | 8.1 | 1.40 (0.88-2.23) | 0.157 |
| Quintile 3 | 44 (2.0) | 9.5 | 1.63 (1.04-2.56) | 0.035 |
| Quintile 4 | 51 (2.4) | 12.0 | 2.08 (1.34-3.23) | 0.001 |
| Quintile 5 | 79 (3.7) | 21.0 | 3.73 (2.47-5.62) | <0.001 |

CI: confidence interval.

**Supplementary Table 8. *Sensitivity analysis 2***- Comparison of the model’s discrimination using the overall C-index and the traditional Harrell’s C-statistic in patients without AF and not receiving anticoagulant therapy at baseline, with occurrence of AF and initiation of anticoagulant therapy as censoring events.

|  | **Overall C-index*** (95%CI) | **Harrell’s C-statistic^†^** (95%CI) |
| --- | --- | --- |
| Stroke model | 0.84 (0.74-0.92) | 0.63 (0.59-0.67) |

* Overall C-index is calculated according to Pencina *et al* method ^21^, as outlined by Liu *et al* ^22^.

^†^ Harrell’s C-statistic is calculated using Harrell *et al* method^11^.

**Supplementary Table 9.** Comparison of the S_2_I_2_N_0-3_ score’s discrimination using the overall C-index and the traditional Harrell’s C-statistic for stroke.

|  | **Overall C-index*** (95%CI) | **Harrell’s C-statistic^†^**(95%CI) |
| --- | --- | --- |
| S_2_I_2_N_0-3_ score | 0.84 (0.76-0.92) | 0.62 (0.59-0.65) |

* Overall C-index is calculated according to Pencina *et al* method ^21^, as outlined by Liu *et al* ^221^.

^†^ Harrell’s C-statistic is calculated using Harrell *et al* method^11^.

‡ Patients with NT-proBNP < 100 pg/mL or > 20000 pg/mL were excluded from analysis. Thus, of 12331 patients with risk score, 12128 patients were evaluated.

**SUPPLEMENTAL FIGURES AND FIGURE LEGENDS**

**Supplementary Figure 1.** Cumulative incidence function plot for stroke for by anticoagulant treatment at baseline.

AF indicates atrial fibrillation; and No., number.

**
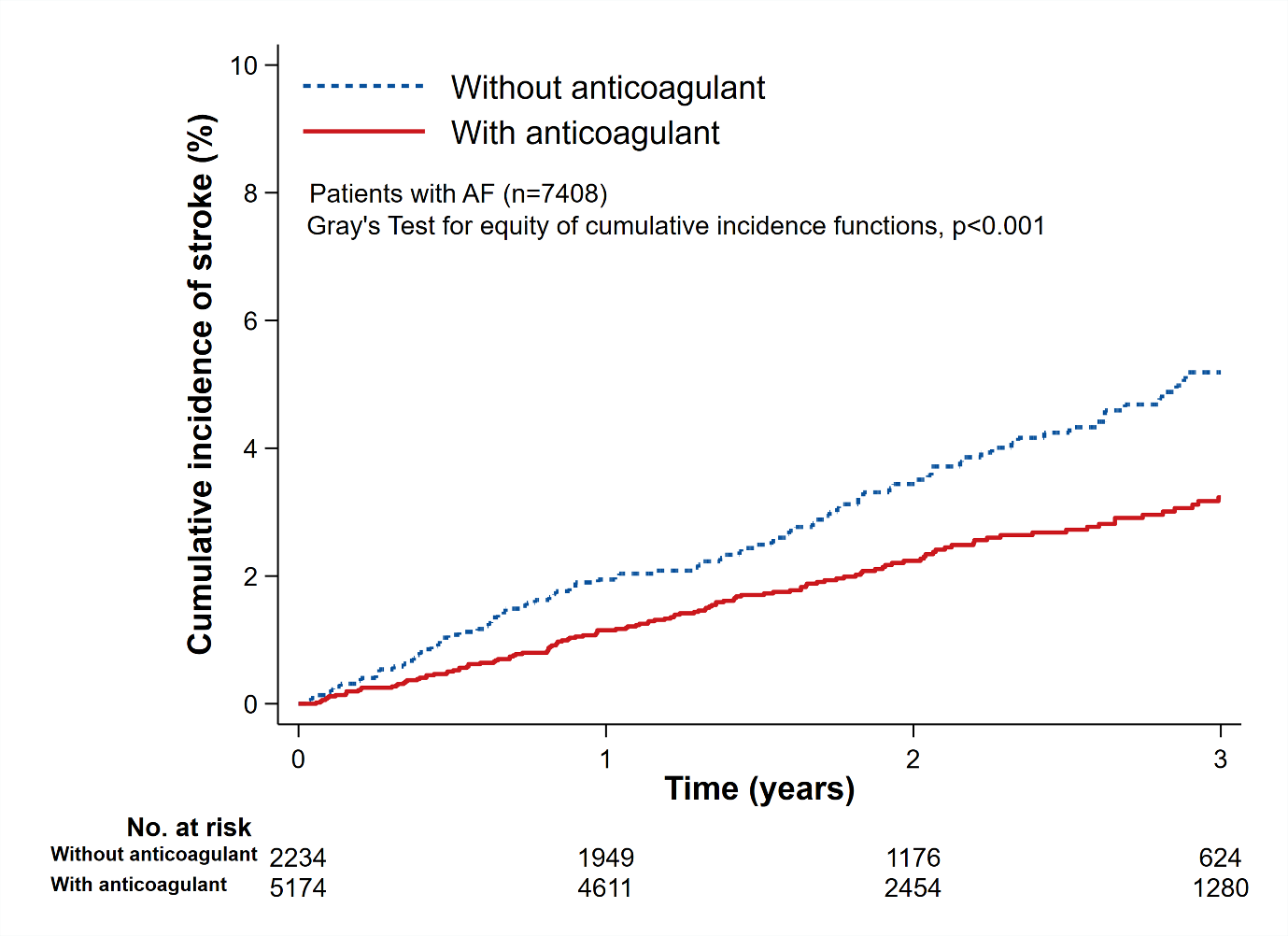
**

**Supplementary Figure 2.** Distribution of the risk score for stroke.

AF indicates atrial fibrillation.


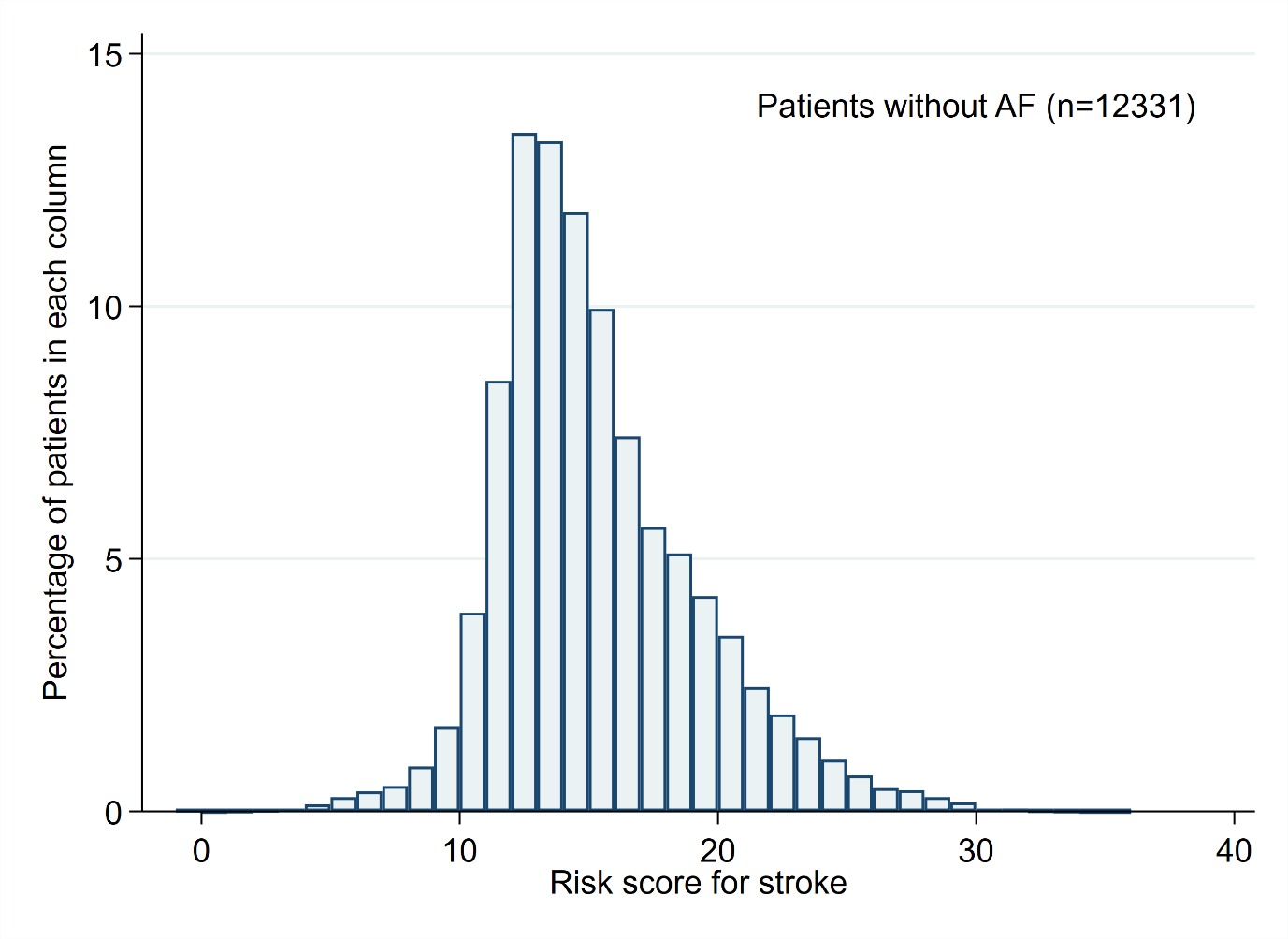


**Supplementary Figure 3. *Sensitivity analysis 1***- Cumulative incidence function plot for ischaemic stroke by quintiles of the risk scores in patients without AF

AF indicates atrial fibrillation; and No., number.


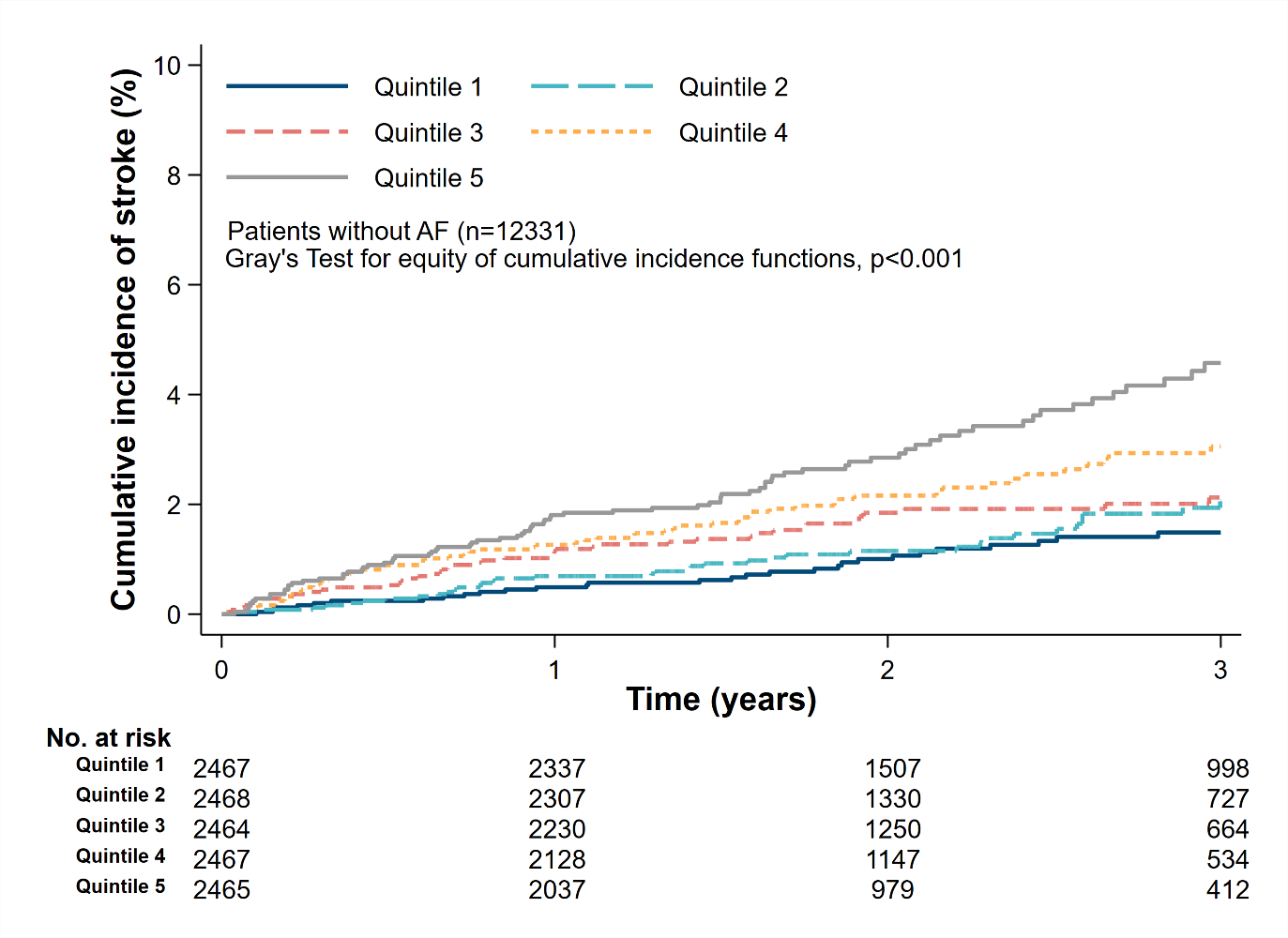


**Supplementary Figure 4. *Sensitivity analysis 1***- Comparison of observed and predicted ischaemic strokes rates after 1 to 3 years for patients categorised by quintiles of risk score.

Calibration plot for 1-year stroke event (A), 2-year stroke event (B), and 3-year stroke event (C). The black circle indicates the observed rate and the black line indicates its range of 95% confidence interval. Each bar is ordered from left to right in descending order of quintiles of risk score. See the supplement for an explanation of how to calculate predicted strokes rates.


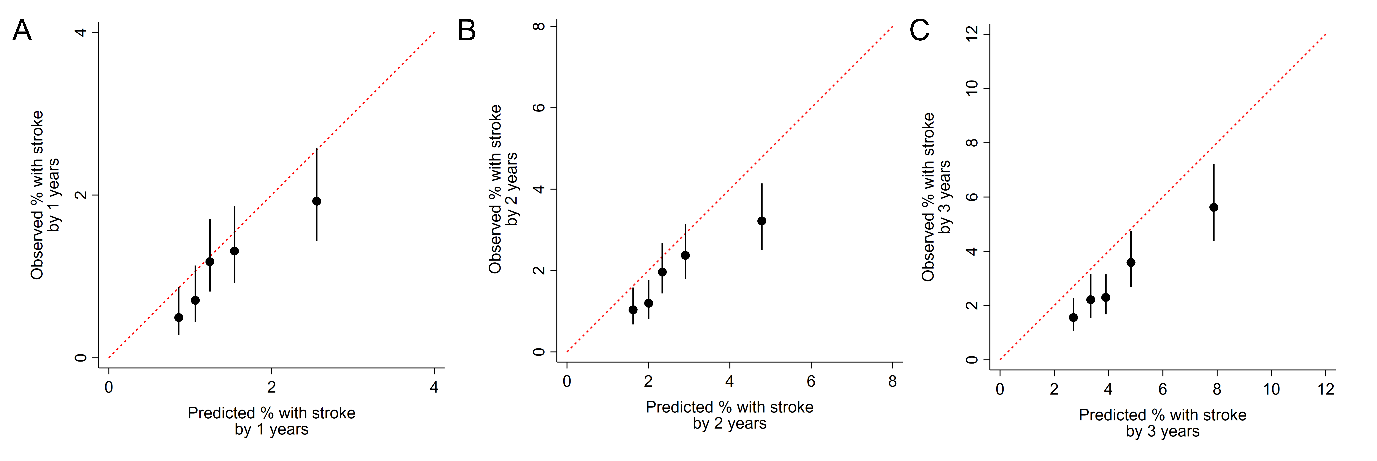


**Supplementary Figure 5. *Sensitivity analysis 2***- Cumulative incidence function plot for stroke by quintiles of the risk scores in patients without AF and not receiving anticoagulant therapy at baseline, with occurrence of AF and initiation of anticoagulant therapy as censoring events

AF indicates atrial fibrillation; and No., number.

**
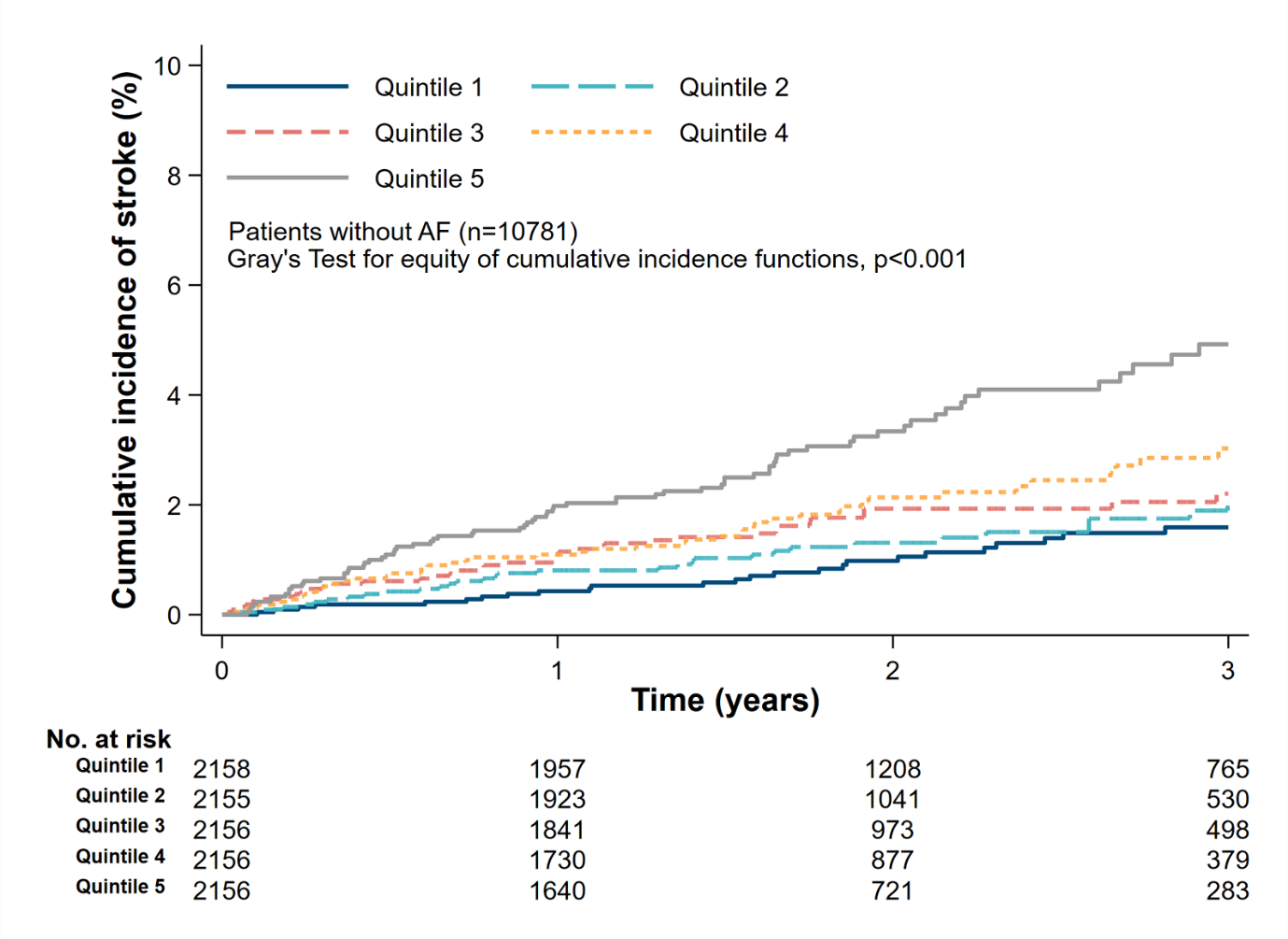
**

**Supplementary Figure 6. *Sensitivity analysis 2***- Comparison of observed and predicted strokes rates after 1 to 3 years for patients, not receiving anticoagulant therapy at baseline, categorised by quintiles of risk score, with occurrence of AF and initiation of anticoagulant therapy as censoring events.

Calibration plot for 1-year stroke event (A), 2-year stroke event (B), and 3-year stroke event (C). The black circle indicates the observed rate and the black line indicates its range of 95% confidence interval. Each bar is ordered from left to right in descending order of quintiles of risk score. See the supplement for an explanation of how to calculate predicted strokes rates.


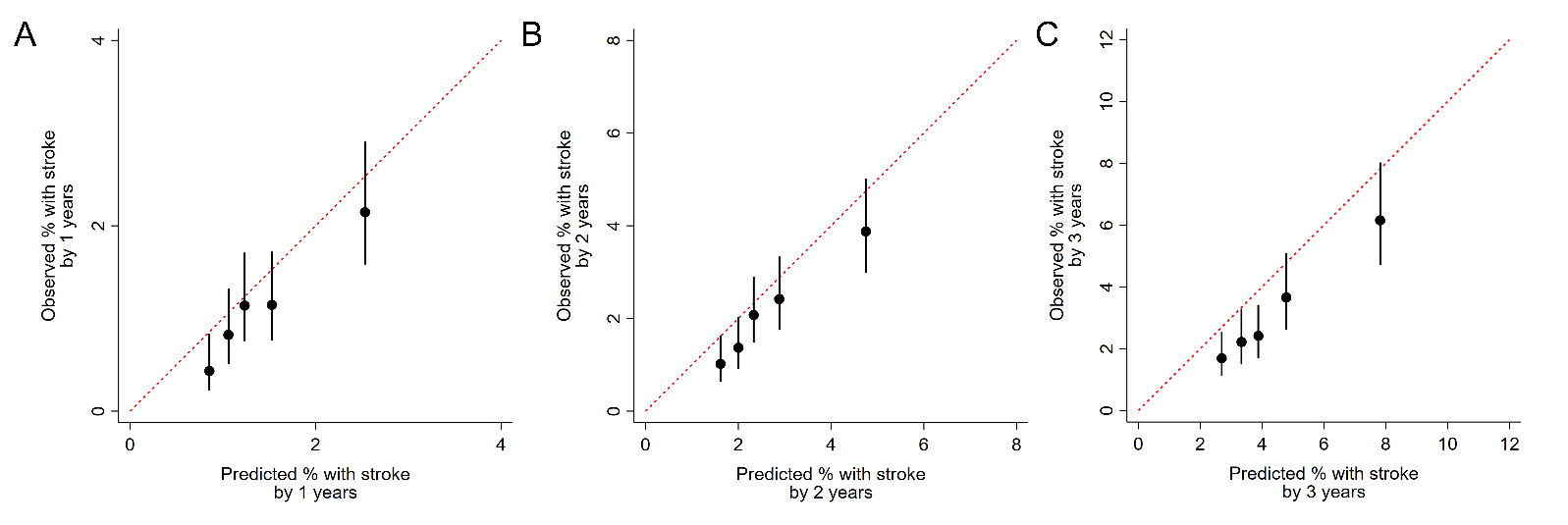


**Appendix.** Examples of calculation of risk score and 1 to 3-year incidence rate for stroke using the model, and S_2_I_2_N_0-3_ score

This example illustrates how to calculate the risk score of stroke and 1 to 3-year incidence rate for stroke in individual patients.

For example, consider a patient aged 70 years who has insulin treated diabetes, history of previous stroke and plasma NT-proBNP measurement of 2000 pg/ml. This patient’s risk score for stroke is: (7.39) + (6.53) + [ln(2000 x 0.1182) × 2.80] = 29.22. (The conversion factor for NT-proBNP is 1 pg/ml = 0.1182 pmol/l.)

1-year incidence rate for stroke is: [1 – 0.9971 ^ exp ( risk score / 10 )] * 100 = 5.3%

2-year incidence rate for stroke is: [1 – 0.9945 ^ exp ( risk score / 10 ) ] * 100 = 9.7%

3-year incidence rate for stroke is: [1 – 0.9908 ^ exp ( risk score / 10 ) ] * 100 = 15.8%

S_2_I_2_N_0-3_ score is: 2 points + 2 points + 2 points = 6 points
